# Self-Labeling Enzyme Tags for Analyses of Translocation of Type III Secretion System Effector Proteins

**DOI:** 10.1128/mBio.00769-19

**Published:** 2019-06-25

**Authors:** Vera Göser, Carina Kommnick, Viktoria Liss, Michael Hensel

**Affiliations:** a Abteilung Mikrobiologie, Universität Osnabrück, Osnabrück, Germany; b CellNanOs–Center for Cellular Nanoanalytics Osnabrück, University of Osnabrück, Osnabrück, Germany; Karlsruhe Institute of Technology (KIT)

**Keywords:** *Salmonella enterica*, cell invasion, facultative intracellular pathogens, live-cell imaging, super-resolution microscopy, type III secretion system

## Abstract

Type III secretion systems (T3SS) are molecular machines in Gram-negative pathogens that translocate effector proteins with central roles in virulence. The analyses of the translocation, subcellular localization, and mode of action of T3SS effector proteins are of central importance for the understanding of host-pathogen interaction and pathogenesis of bacterial infections. The analysis of translocation requires dedicated techniques to address the temporal and spatial dynamics of translocation. Here we describe a novel approach to deploy self-labeling enzymes (SLE) as universal tags for localization and tracking of translocated effector proteins. Effector-SLE fusion proteins allow live-cell imaging of translocation by T3SS, superresolution microscopy, and single-molecule tracking of effector motility in living host cells. We describe the application of the approach to T3SS effector proteins for invasion and intracellular lifestyle of Salmonella enterica serovar Typhimurium and to a T3SS effector of Yersinia enterocolitica. The novel approach enables analyses of the role of T3SS in host-pathogen interaction at the highest temporal and spatial resolution, toward understanding the molecular mechanisms of their effector proteins.

## Abstract (precis)

Type III secretion systems mediate translocation of effector proteins into mammalian cells. These proteins interfere with host cell functions, being main virulence factors of Gram-negative pathogens. Analyses of the process of translocation, the subcellular distribution, and the dynamics of effector proteins in host cells have been hampered by the lack of suitable tags and detection systems. Here we describe the use of self-labeling enzyme tags for generation of fusions with effector proteins that are translocated and functional in host cell manipulation. Self-labeling reactions with cell-permeable ligand dyes are possible prior to or after translocation. We applied the new approach to superresolution microscopy for effector protein translocation. For the first time, we show the dynamic properties of effector proteins in living host cells after translocation by intracellular bacteria. The new approach of self-labeling enzyme tags fusions will enable analyses of type III secretion system effector proteins with new dimensions of temporal and spatial resolution.

## INTRODUCTION

Almost all Gram-negative pathogens deploy protein translocation systems for the direct delivery of effector proteins into specific compartments of a target host cell (see reference 1 for an overview). The best-studied translocation systems are type III secretion systems (T3SS), type IV secretion systems (T4SS), and type VI secretion systems (T6SS). Key functions of these systems for the pathogenesis of bacterial infections have been documented for various Gram-negative pathogens. A common feature of translocation systems is the delivery of one or multiple effector proteins into mammalian target cells (T3SS, T4SS, and T6SS), or other bacterial cells (T6SS). Unraveling the interference of effector proteins with normal functions of the target cells is a key step to understand bacterial pathogenesis. Apart from analyses of the biochemical functions of effector proteins, analyses of mechanisms and kinetics of translocation, the subcellular distribution of effector proteins, and their molecular interaction with host cell structures are important elements in understanding the bacterial manipulation of host cell functions.

To localize effector proteins in host cells, direct detection by immunolabeling of the effector protein or recombinantly introduced epitope tags has been frequently used. This approach, however, is restricted to fixed, permeabilized cells and cannot address the kinetics and dynamics of effector translocation and distribution. Use of fluorescent proteins (FP) has revolutionized cell biology by allowing analyses of FP-tagged proteins in living cells (2). FP also allow live-cell imaging of bacteria for a wide range of applications, for example, to follow dynamics of cytoskeletal elements in prokaryotes. However, FP tags have not been useful to investigate effector translocation, and it has been shown that the formation of stable tertiary structures in folded FP blocks the process of translocation by T3SS. Bimolecular fluorescence complementation has been successfully applied by tagging effector proteins with a short domain of an FP and expressing the remaining portion within the host cell (3). Other tags have been used to follow effector protein translocation, such as the tetracysteine tag in complex with biarsenic dyes (4, 5).

A series of enzymes is compatible with translocation if fused to effector proteins, and enzymes such as β-lactamase, adenylate cyclase, Cre-Lox, and luciferases have been used to quantify translocation of effectors into target cells. However, these enzymes do not allow molecular imaging. Alternative enzyme tags are self-labeling enzyme (SLE) tags, such as HaloTag (6) or SNAP-tag or CLIP-tag (7, 8). SLEs catalyze reactions with specific substrates which then remain bound to the SLE. By fusing ligands such as fluorochromes to SLE substrates, self-labeling reactions may introduce specific fluorescent labeling to fusion proteins. We have previously introduced self-labeling enzyme tags as versatile markers for labeling subunits of protein secretion systems (9), enabling superresolution microscopy (SRM) and localization and tracking microscopy (TALM). We speculated that genetically encoded SLE might also be useful markers to label translocated effector proteins of T3SS in order to follow the fate of these proteins from translocation to final position in host cells and for analysis in living host cells.

In this study, we investigated the application of three commonly used SLEs, HaloTag, SNAP-tag, and CLIP-tag for tagging effector proteins of T3SS of Salmonella enterica serovar Typhimurium and Yersinia enterocolitica. We observed that T3SS effector protein-SLE fusions are translocated into host cells, occupy proper subcellular localization, and are functional in manipulating host cell functions. Specific labeling of effector protein-SLE fusion proteins with SRM-compatible dyes allowed imaging of the subcellular localization below the diffraction limit and single-molecule tracking of effector proteins in living host cells. Here we evaluate the use of different SLE tags and demonstrate the applicability of SLE tags for new high-resolution and live-cell imaging applications in infection biology.

## RESULTS

### Self-labeling enzymes as genetically encoded tags for analyses of T3SS effector protein translocation.

We set out to generate a system that allows analyses of translocated effector proteins in living host cells. Since SLEs are versatile tags for multiple applications involving protein tagging and localization, we considered them as potential tags for bio-orthogonal and live-cell-compatible labeling of effector proteins. To test their potential use, we selected the commonly used SLE HaloTag, SNAP-tag, and CLIP-tag.

Salmonella enterica serovar Typhimurium (STM) deploys two distinct T3SS during pathogenesis. The *Salmonella* pathogenicity island 1 (SPI1)-encoded T3SS translocates a set of preformed effector proteins mediating host cell invasion, while the *Salmonella* pathogenicity island 2 (SPI2)-encoded T3SS is synthesized, assembled, and activated by STM residing in the *Salmonella*-containing vacuole of infected host cells (reviewed in reference 10). The effector proteins of the SPI2-T3SS, among other functions, interfere with the organization and trafficking of endosomal membrane vesicles of the host cell. Due to distinct roles on host-pathogen interaction, we considered SPI1-T3SS and SPI2-T3SS effector proteins as interesting targets for evaluating SLE fusions.

Since T3SS effector proteins contain N-terminal recognition sequences for the T3SS and N-terminal chaperone-binding domains, protein fusions are commonly introduced at the C-terminus. Accordingly, we generated genetic fusions of various effector proteins, a linker sequence, either HaloTag, SNAP-tag, or CLIP-tag, and a hemagglutinin (HA) epitope tag to allow uniform detection of fusion proteins. The resulting fusion proteins were expressed by chromosomal genes in their native location or by expression cassettes on low-copy-number vectors.

We analyzed synthesis of fusion proteins and secretion *in vitro*, and Fig. S1 in the supplemental material shows examples for fusions of STM SPI1-T3SS effector SopE and Yersinia enterocolitica T3SS effector YopM to various tags. The various fusions were synthesized and SopE and YopM fusions to SNAP-tag or CLIP-tag were secreted in a T3SS-dependent manner. For various SPI2-T3SS effector proteins, fusion proteins with SLE tags were synthesized under SPI2-T3SS-inducing conditions *in vitro* (data not shown). Secretion *in vitro* was not analyzed for these proteins, since we focused on translocation by intracellular STM.

10.1128/mBio.00769-19.1 FIG S1 Synthesis and secretion of SLE-tagged effector proteins. WT STM, the SPI1-T3SS-deficient Δ*invC* strain, or Y. enterocolitica WA-C harboring plasmids for the expression of *sopE* or *yopM* to HA tag or various SLEs was used. For detection of SopE fusion proteins (A and B), cultures were grown with aeration at 37°C for 6 h. For detection of YopM fusion proteins (C and D), cultures were grown with aeration at 37°C for 90 min and secretion was induced by addition of 15 mM MgCl, 5 mM EGTA, and 0.2% glucose and incubation for 2 h. Bacterial pellets (B and D) and supernatants (A and C) were collected. Protein in supernatants was precipitated by addition of 20% trichloroacetic acid; samples were processed for Western blot analyses of the HA epitope tag. DnaK was used as a control for loading and cytosolic contamination of supernatant fractions. Detected bands represent SopE-HA (28 kDa), SopE-HaloTag-HA (62 kDa), SopE-SNAP-tag-HA (48 kDa), and SopE-CLIP-tag-HA (48 kDa) from supernatants (A) and cell lysates (B). Detected bands represent YopM-HaloTag-HA (89 kDa), YopM-SNAP-tag-HA (77 kDa), and YopM-CLIP-tag::HA (77 kDa) in supernatants (C) and cell lysates (D). Download FIG S1, TIF file, 2.2 MB. Copyright © 2019 Göser et al. 2019 Göser et al. This content is distributed under the terms of the Creative Commons Attribution 4.0 International license.

### Effector-SLE fusions proteins are translocated by T3SS and functional as virulence proteins in *Salmonella*.

We first determined if SLE-tagged effector proteins are functional as virulence factors of STM. A mutant strain deficient in SPI1-T3SS effector proteins SipA, SopA, SopB, SopE, and SopE2 is highly attenuated in invasion of epithelial cells (11). Complementation of this strain with single effector *sopE* or *sipA*, fused to either SNAP-tag or CLIP-tag, resulted in highly increased invasion (Fig. S2A). Invasion was not affected by labeling with SLE ligands prior to infection (Fig. S2B). In contrast, fusion proteins of *sopE* or *sipA* and HaloTag were less efficient, as only partial restoration of invasiveness was observed (data not shown).

10.1128/mBio.00769-19.2 FIG S2 SLE fusions to SPI1-T3SS effector proteins are functional in STM invasion. Invasion of HeLa cells by STM was determined by gentamicin protection assays. (A) HeLa cells were infected with WT STM, the Δ*invC* strain defective in the SPI1-T3SS, strain Δ5 with deletion of SPI1-T3SS effector genes *sipA*, *sipB*, *sopD*, *sopE*, and *sopE2*, or the Δ5 strain harboring plasmids for expression s*opE*::SNAP-tag::HA, *sopE::*CLIP-tag::HA, *sipA*::SNAP-tag::HA, or *sipA:*:CLIP-tag::HA as indicated. After infection at an MOI of 1, medium with 100 μg · ml^−1^ of gentamicin was used to kill extracellular bacteria for 1 h. Intracellular CFU counts were determined 1 h postinfection, and invasion is the ratio of CFU recovered at 1 h and CFU in inoculum. Depicted are means and SDs of triplicates, and data for one of three biological replicates are shown. (B) Strains harboring plasmids for expression of s*opE*::SNAP-tag::HA, *sopE::*CLIP-tag::HA, *sipA*::SNAP-tag::HA, or *sipA:*:CLIP-tag::HA were left untreated (− ligand) or labeled prior to infection with 1 μM SNAP-Cell TMR-Star or CLIP-Cell TMR-Star during the last 30 min of the 3.5-h subculture (+ ligand). Invasion of HeLa cells was determined as described for panel A. Shown are means and SDs of at least triplicates of one biological replicate. Statistical analyses were performed by Student’s *t* test (SigmaPlot 13.0; Systat), and significances are indicated as follows: n.s., not significant; **, *P* < 0.01; and ***, *P* < 0.001. Download FIG S2, TIF file, 0.1 MB. Copyright © 2019 Göser et al. 2019 Göser et al. This content is distributed under the terms of the Creative Commons Attribution 4.0 International license.

Mutant strains deficient in SPI2-T3SS effector protein SifA or SseF are reduced in intracellular proliferation and fully or partly impaired in remodeling of the host cell endosomal system, usually resulting in formation of *Salmonella*-induced filaments (SIF) (12, 13). Complementation of *sifA* or *sseF* mutant strains with plasmids for expression of *sifA*::HaloTag::HA or *sseF*::HaloTag::HA restored intracellular proliferation (Fig. S3A) and the ability to induce SIF (Fig. S3B). The fusions of *sifA* or *sseF* to SNAP-tag or CLIP-tag failed to complement the intracellular replication of the corresponding mutant strains.

10.1128/mBio.00769-19.3 FIG S3 SLE fusions to SPI2-T3SS effector proteins are functional in intracellular pathogenesis of STM. (A) Intracellular replication of STM was determined by gentamicin protection assays. RAW264.7 macrophages were infected with WT STM, Δ*ssaV*, Δ*sseF*, or Δ*sifA* strains, or mutant strains expressing *sseF*::HaloTag::HA or *sifA*::HaloTag::HA as indicated. After infection at an MOI of 1, medium with 100 μg · ml^−1^ of gentamicin was used to kill extracellular bacteria for 1 h, followed by incubation with medium containing gentamicin at 10 μg · ml^−1^. Intracellular CFU counts were determined 2 and 16 h postinfection, and intracellular replication is the ratio of CFU at 16 and 2 h. (B) For analyses of induction of SIF, HeLa cells stably expressing LAMP1-meGFP were infected with WT STM, Δ*ssaV*, Δ*sseF*, or Δ*sifA* strains, or mutant strains expressing *sseF*::HaloTag::HA or *sifA*::HaloTag::HA as indicated. After infection at an MOI of 75, cells were incubated for 8 h under standard cell culture conditions, washed, and fixed with 3% PFA. Immunostaining was performed for STM O antigen. For each condition 100 cells were counted and infected cells were checked for SIF formation. Depicted are means and SDs of triplicates, and data for one of three biological replicates are shown. Statistical analyses were performed by Student’s *t* test (SigmaPlot 13.0; Systat), and significances are indicated as follows: n.s., not significant; *, *P* < 0.05; **, *P* < 0.01; and ***, *P* < 0.001. Download FIG S3, TIF file, 0.2 MB. Copyright © 2019 Göser et al. 2019 Göser et al. This content is distributed under the terms of the Creative Commons Attribution 4.0 International license.

We tested the translocation of T3SS effector-SLE fusion proteins. For this, the amounts of translocated effector proteins and the subcellular localization were compared to those of effector proteins labeled with an M45 or HA epitope tag. SipA, SopB, and SopE are representative effector proteins of the SPI1-T3SS. The translocation by STM with these effector proteins tagged with either the HA epitope tag only or HaloTag-HA, SNAP-tag-HA, or CLIP-tag-HA was analyzed (Fig. S4A to D). We observed that HaloTag-HA-labeled effector proteins were not or only poorly translocated, while immunostaining for SNAP-tag-HA or CLIP-tag-HA fusion proteins in host cells was comparable to that for effector proteins labeled with the HA tag only.

10.1128/mBio.00769-19.4 FIG S4A (A to D) Translocation of effector proteins fused to various SLE. For analyses of translocation of SPI1-T3SS effector proteins, infection was performed with WT STM harboring plasmids for the expression of *sipA* (A), *sopB* (B), or *sopE* (C) fused to HaloTag, SNAP-tag, or CLIP-tag as indicated. All effector proteins comprised a C-terminal HA epitope tag for immunodetection of translocated protein. HeLa cells constitutively expressing LifeAct-GFP (green) were used for infection. WT STM with empty plasmid was used as a negative control (D). Download FIG S4A, JPG file, 2.7 MB. Copyright © 2019 Göser et al. 2019 Göser et al. This content is distributed under the terms of the Creative Commons Attribution 4.0 International license.

For effector proteins of the SPI2-T3SS, we subjected PipB2, SifA, SseF, and SseJ to a similar evaluation (Fig. S4E to I). For these effector proteins, the HaloTag or SNAP-tag fusion proteins were efficiently translocated, while the CLIP-tag-labeled effector proteins gave only poor or no signals after immunostaining for translocated effector proteins.

10.1128/mBio.00769-19.11 FIG S4B (E to J) Translocation of effector proteins fused to various SLEs. For translocation of SPI2-T3SS effector proteins, infection was performed with WT STM harboring plasmids for the expression of *pipB2* (E), *sifA* (F), *sseF* (G), or *sseJ* (H) fused to HaloTag, SNAP-tag, or CLIP-tag as indicated. All effector proteins comprised a C-terminal HA epitope tag for immunodetection of translocated protein. HeLa cells constitutively expressing LAMP1-GFP (green) were used for infection. WT STM with empty plasmid was used as a negative control (I). For translocation of Y. enterocolitica effector protein YopM (J), infection of HeLa cells constitutively expressing LAMP1-GFP (green) was performed with Y. enterocolitica WA-C(pTTSS) harboring plasmids for the expression of *yopM* fused to HaloTag, SNAP-tag, or CLIP-tag as indicated. Y. enterocolitica WA-C(pTTSS) with empty plasmid was used as a negative control. All effector proteins comprised a C-terminal HA epitope tag for immunolabeling of translocated protein. After fixation and permeabilization, immunolabeling of STM or Y. enterocolitica (blue) and HA tag (red) was performed. Scale bars, 10 μm. Download FIG S4B, JPG file, 2.9 MB. Copyright © 2019 Göser et al. 2019 Göser et al. This content is distributed under the terms of the Creative Commons Attribution 4.0 International license.

To investigate the application to T3SS effector proteins of additional pathogens, we selected YopM of Y. enterocolitica. Immunostaining of translocated fusion proteins indicated that YopM-CLIP-tag could be detected, while HaloTag or SNAP-tag fusion proteins resulted in only very weak signals in host cells (Fig. S4J).

Taken together, these observations show that effector proteins fused to SLE HaloTag, SNAP-tag, or CLIP-tag are translocated by T3SS into host cells. However, the signal intensities varied considerably between combinations of effector classes and type of SLE used for fusion. Thus, initial validation of compatibility of effector proteins with SLE prior to further applications is highly recommended.

**FIG 1:**
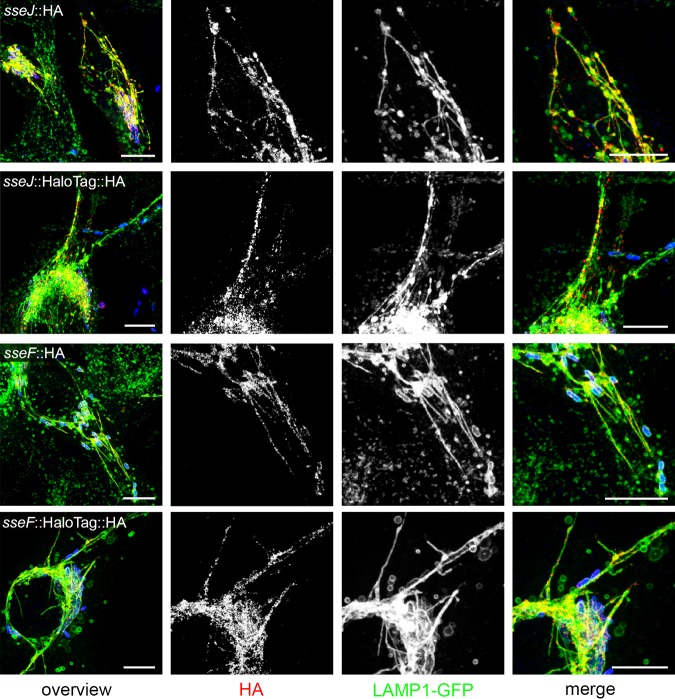
Effect of HaloTag fusion on translocation and subcellular distribution of T3SS effector proteins. STM harboring plasmids for the expression of *sseJ*::HA, *sseJ*::HaloTag::HA, *sseF*::HA, or *sseF*::HaloTag::HA were used for infection of HeLa cells constitutively expressing LAMP1-GFP (green). Sixteen hours postinfection, cells were fixed, permeabilized by saponin, and immunolabeled for the HA epitope tag (red). Scale bars, 10 μm.

We observed that fusion of HaloTag to various SPI2-T3SS effector proteins was fully compatible with translocation into host cells. SseF and SseJ are representative SPI2-T3SS effector proteins that show a distinct localization at the *Salmonella*-containing vacuole and SIF after translocation, as well as a prominent colocalization with late endosomal/lysosomal glycoprotein LAMP1. In this study, we routinely used HeLa cells constitutively expressing LAMP1-monomeric enhanced green fluorescent protein (meGFP) as host cells for infection. The translocated effector proteins with SLE fusions showed the same subcellular localization as effector proteins with short epitope tags (Fig. 1). Since SPI1-T3SS effector proteins show a more diffuse distribution in host cells, we did not analyze the effect of SLE tags on distribution of these proteins.

### Labeling of effector SLE fusion proteins with cell-permeable ligands allows detection of translocated effector proteins in living host cells.

**FIG 2:**
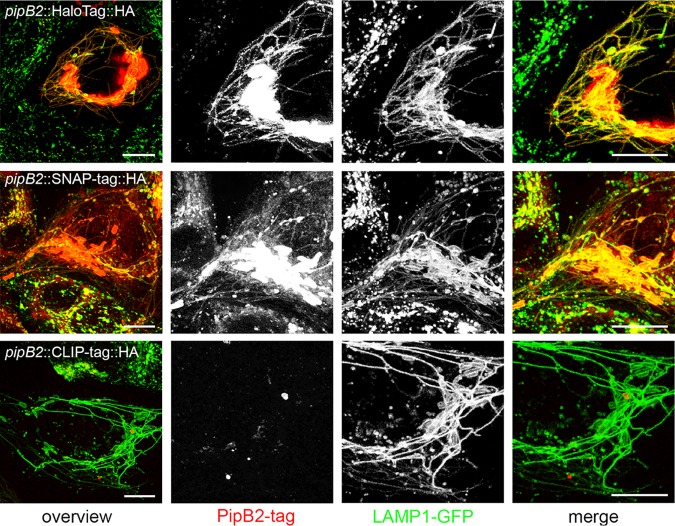
Labeling specificity of SLE in live-cell imaging. HeLa cells stably expressing LAMP1-meGFP were seeded in 8-well chamber slides. The next day, cells were infected with STM Δ*pipB2* harboring plasmids for expression of *pipB2*::HaloTag::HA, *pipB2*::SNAP-tag::HA, or *pipB2*::CLIP-tag::HA at an MOI of 50. Live-cell imaging was performed at 16 h postinfection. Labeling reactions were performed directly before imaging using the respective SLE ligand for 30 min at 37°C. Representative STM-infected host cells exhibiting SIF formation were selected for live-cell imaging. Scale bars, 10 μm.

The reaction with and covalent binding of cognate ligands is a critical requirement for the application of effector-SLE fusion proteins. We compared the labeling of an SPI2-T3SS effector protein fused to either HaloTag, SNAP-tag, or CLIP-tag with the corresponding ligand coupled to tetramethylrhodamine (TMR) after translocation (Fig. 2). We performed live-cell imaging of infected HeLa LAMP1-meGFP cells. In line with the small amounts of PipB2-CLIP-tag-HA observed (Fig. S4E), the TMR signals for labeled CLIP-tag were very low and predominantly localized to intracellular STM. Strong TMR signal intensities were observed for PipB2-HaloTag-HA and PipB2-SNAP-tag-HA. The labeling colocalized with the tubular LAMP1-positive SIF, similar to the observation for HA tag immunolabeling of PipB2-HA or PipB2-SLE-HA. While background signals were virtually absent for HaloTag ligand (HTL)-TMR and signals were restricted to infected host cells, significant background signals in infected and noninfected cells were observed for SNAP-tag ligand SNAP-Cell TMR-Star. Strong labeling of intracellular bacteria was observed, indicating labeling of the pool of effector proteins remaining in the bacterial cytosol.

**FIG 3:**
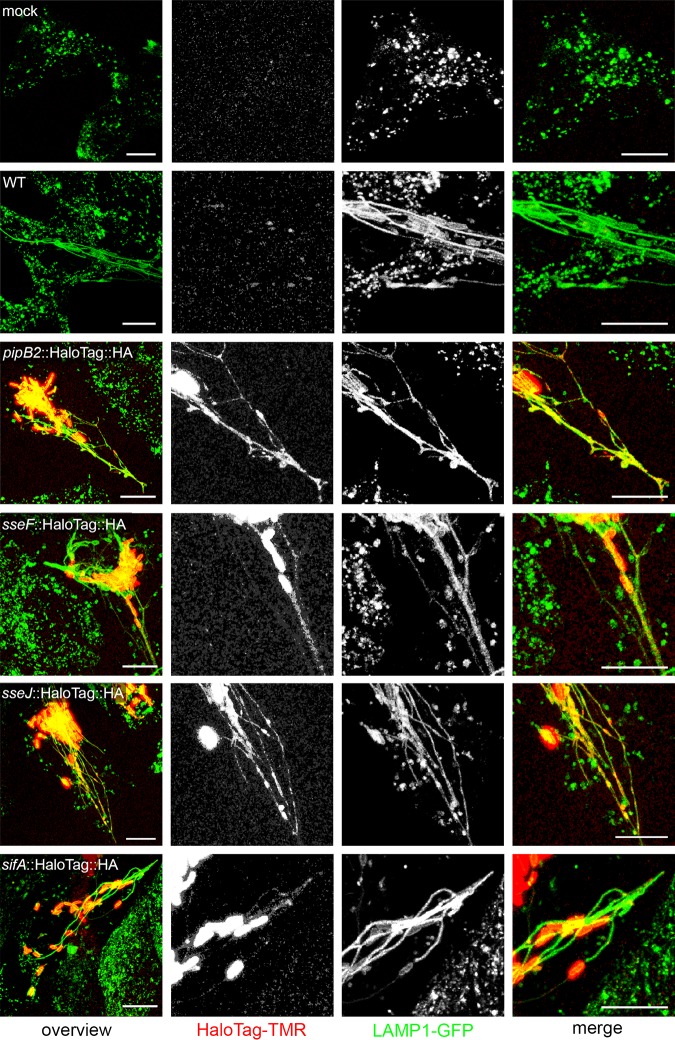
HaloTag as a genetically encoded fluorescent marker for live-cell imaging of *Salmonella* SPI2-T3SS effector proteins. HeLa cells stably expressing LAMP1-meGFP were seeded in 8-well chamber slides. The next day, cells were mock infected or infected at an MOI of 50 with WT STM or mutant strains expressing the respective effector as a HaloTag fusion, i.e., Δ*pipB2* [*pipB2*::HaloTag::HA], Δ*sseF* [*sseF*::HaloTag::HA], Δ*sseJ* [*sseJ*::HaloTag::HA], and Δ*sifA* [*sifA*::HaloTag::HA] strains. Live-cell imaging was performed at 16 h postinfection. Labeling reactions were performed directly before imaging using HTL-TMR with a final concentration of 1 μM for 30 min at 37°C. Scale bars, 10 μm.

We performed live-cell imaging of further SPI2-T3SS effector proteins fused to HaloTag and labeled with HTL-TMR in living host cells after infection (Fig. 3). Similar to the case with PipB2-HaloTag-HA, preferential localization of SseF-HaloTag-HA, SseJ-HaloTag-HA, and SifA-HaloTag-HA to *Salmonella*-containing vacuole and SIF membranes was observed, while nonspecific background signals were negligible. Similar results were obtained for various additional SPI2-T3SS effector proteins.

From these data, we conclude that SLE tags are compatible with specific detection of translocated effector proteins in living host cells. In addition to compatibility of SLE with the T3SS of a given effector protein, the efficiency of the self-labeling reaction and removal of nonbound ligand should be considered for selection of the most suitable tagging strategy. For SPI2-T3SS effector proteins, HaloTag gave the best results.

### Prelabeled effector proteins are translocated.

TMR-conjugated ligands for SLE are cell-permeable, and we previously demonstrated the labeling of various SLE-tagged proteins in STM (9, 14). The strong labeling of effector-SLE fusion proteins in bacterial cells observed in this study suggests that the SLE moiety is enzymatically active in self-labeling of preformed proteins in the bacterial cytosol. We tested if such preformed and self-labeled effector-SLE fusions can be translocated into host cells. Since SPI2-T3SS effector proteins are synthesized after entry into cells and do not allow to address the time point of labeling, we investigated SopE as a representative SPI1-T3SS effector protein. These effector proteins are preformed in bacteria prior to infection of host cells.

**FIG 4:**
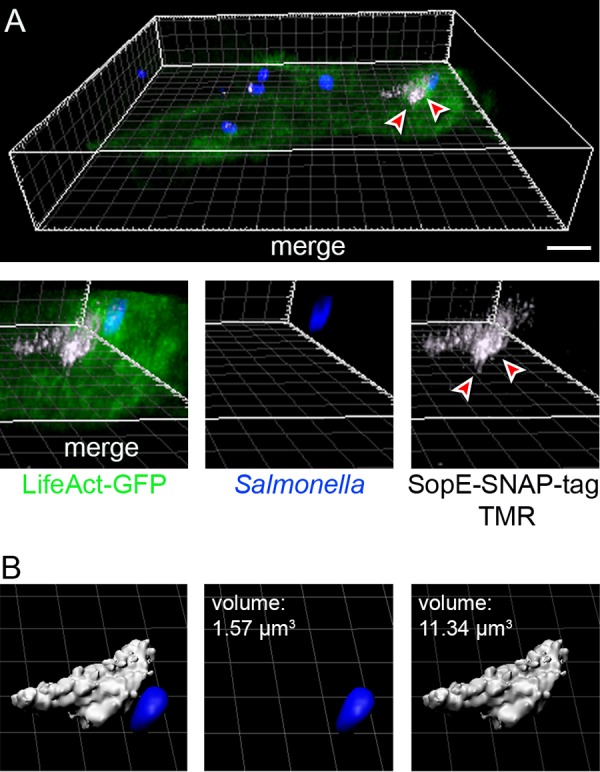
Translocation of prelabeled effector proteins. HeLa cells stably expressing LifeAct-GFP (green) were seeded in 8-well chamber slides. STM expressing *sopE*::SNAP-tag::HA was subcultured to induce invasion genes and synthesis of SPI1-T3SS effector proteins. Bacteria were incubated with 1 μM CellStar-TMR (red) for 30 min at 37°C and used to infected host cells. After infection, cells were fixed and STM was immunolabeled for O antigen (blue). (A) An area of TMR-labeled translocated SopE-SNAP-tag is indicated by arrowheads. Projections of the data set were rendered using Imaris; an animated three-dimensional (3D) projection is available under https://myshare.uni-osnabrueck.de/f/7c7152b73af64e37a465/. (B) Volume quantifications of STM and the SopE-SNAP-tag positive area were performed in Imaris. Scale bars, 10 μm.

Bacteria were subcultured to induce invasiveness and labeled with ligand for 30 min prior to infection or during the infection period (Fig. S5). Adhesion of STM to host cells and initiation of membrane ruffles were observed, indicating ongoing invasion. Signals for TMR-labeled SopE-SNAP-tag and SopE-CLIP-tag were detected for cells incubated with the ligand prior to or during infection. Membrane ruffles were also positive for TMR fluorescence, and the signals were located within a volume of 11.34 μm^3^ in a representative host cell adjacent to one invading STM (Fig. 4). Signal intensity for CLIP-tag was weaker than for SNAP-tag. For Y. enterocolitica YopM, labeling during infection gave no detectable signal, while prelabeling resulted in signals for YopM-CLIP-tag with diffuse distribution in infected cells (Fig. S5). These observations indicate that self-labeling reactions of SLE can be performed in bacteria and that labeled effector-SLE fusion proteins assume a conformation that is compatible with translocation by the T3SS. We performed live-cell imaging with prelabeled STM expressing *sopE*::SNAP-tag::HA. At sides of contact of STM to host cells and SPI1-T3SS-induced F-actin formation, TMR signals were found in the host cell, clearly distant to the invading bacterial cell (Movie S1).

10.1128/mBio.00769-19.6 FIG S5 Translocation of effector proteins labeled prior to or during infection. (A and B) HeLa cells stably expressing LifeAct-GFP (green) were seeded in 8-well chamber slides. WT STM or strains expressing *sopE*::SNAP-tag::HA or *sopE*::CLIP-tag::HA were subcultured to induce invasion genes and synthesis of SPI1-T3SS effector proteins. (C and D) HeLa cells stably expressing LAMP1-GFP (green) were seeded in 8-well chamber slides and infected with Y. enterocolitica WA-C(pTTSS) without or with plasmid for expression of *yopM*::CLIP-tag::HA as for Fig. S4J. (A and C) During infection, 1 μM CellStar-TMR was added to infected cells. (B and D) Bacteria were incubated with 1 μM CellStar-TMR (red) for 30 min at 37°C prior to infection and used to infect host cells as shown for Fig. 4. After infection, cells were fixed and STM or Y. enterocolitica was immunolabeled for O antigen (blue). Scale bars, 10 μm. Download FIG S5, JPG file, 2.4 MB. Copyright © 2019 Göser et al. 2019 Göser et al. This content is distributed under the terms of the Creative Commons Attribution 4.0 International license.

10.1128/mBio.00769-19.9 MOVIE S1 Live-cell time-lapse microscopy of invasion of HeLa cells expressing LifeAct-GFP (green) by STM expressing *sopE*::SNAP-tag::HA prelabeled with SNAP-tag ligand CellStar-TMR (white). Note the appearance of TMR-positive areas in host cells at sides to ongoing invasion. Download Movie S1, MPG file, 1.1 MB. Copyright © 2019 Göser et al. 2019 Göser et al. This content is distributed under the terms of the Creative Commons Attribution 4.0 International license.

### Superresolution microscopy of translocated effector proteins.

The self-labeling reaction of SLE tags allows covalent coupling of cell-permeable fluorochromes with blinking properties to fusion proteins. These fluorochromes have been used for stochastic superresolution microscopy (SRM), for example, to localize bacterial secretion systems (9). Since effector-SLE fusion proteins are efficiently translocated and labeled with TMR-conjugated ligands into host cells, we anticipated that TMR-labeled effector-SLE fusion proteins could allow SRM of translocated effector proteins.

**FIG 5:**
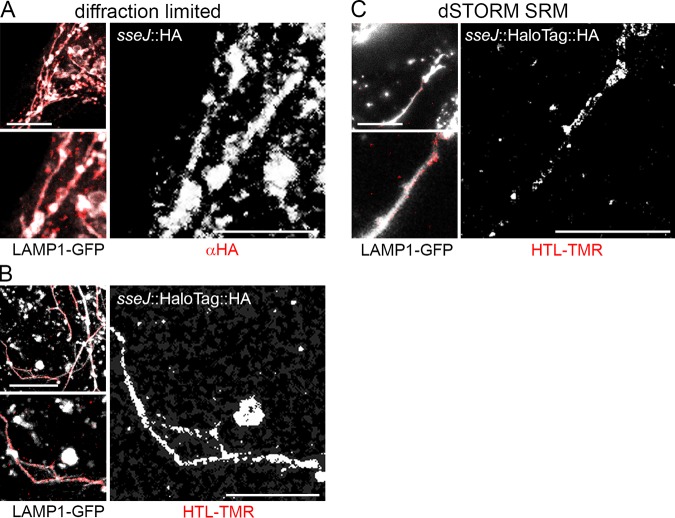
HaloTag as a genetically encoded marker for superresolution microscopy of effector protein localization. HeLa cells stably expressing LAMP1-GFP were infected with an STM *sseJ* mutant strain expressing *sseJ*::HA (A) or *sseJ*::HaloTag::HA (B and C) at an MOI of 75. Following incubation for 8 h, infected cells were fixed. (A) For comparison, confocal microscopy was performed after immunostaining of the HA tag. (B and C) Labeling reactions using HTL-TMR were performed directly before fixation. For SRM by dSTORM imaging, cells were incubated in a buffer containing 100 mM β-mercaptoethylamine, 4.5 mg · ml^−1^ of d-glucose, 40 μg · ml^−1^ of catalase, and 0.5 mg · ml^−1^ of glucose-oxidase, and maximum laser power was used for excitation. Shown are representative diffraction-limited confocal laser-scanning microscopy (A and B) and dSTORM SRM (C) images. The SRM image was rendered from single-emitter localizations obtained within 500 frames. Scale bars, 10 and 5 μm in overviews and details, respectively. SRM of effector-SLE fusions for *pipB2*, *sseF*, *sifA*, and a negative control (WT STM without SLE) are shown in Fig. S6.

We used SPI2-T3SS effector proteins labeled after translocation into HeLa cells expressing LAMP1-GFP for localization of the *Salmonella*-containing vacuole and SIF membranes. SRM was performed for PipB2, SseF, SseJ, and SifA using direct stochastical optical reconstruction microscopy (dSTORM), a pointillist approach that requires blinking properties of fluorochromes in order to record hundreds of individual frames for calculation of improved localization. As anticipated from previous investigations, these effector proteins were predominantly associated with LAMP1-positive membranes, mainly present in SIF (Fig. 5 and Fig. S6). A lower number of signals was associated with LAMP1-positive vesicles that were not part of *Salmonella*-containing vacuole or SIF. We compared conventional diffraction-limited confocal microscopy and SRM (Fig. 5) and observed highly improved localization of subcellular positions of translocated effector in dSTORM images.

10.1128/mBio.00769-19.7 FIG S6 SRM for plasmid-encoded or chromosome-encoded effector-HaloTag fusions. (A) HeLa cells stably expressing LAMP1-meGFP were infected with STM strains harboring plasmids for expression of *sseJ*, *pipB2*, *sseJ*, or *sifA* to HaloTag::HA as indicated at an MOI of 75. (B) HeLa cells stably expressing LAMP1-meGFP were infected with STM strains expressing chromosomal fusion of *pipB2*, *sseJ*, or *sifA* to HaloTag::HA as indicated at an MOI of 75. SRM of effector-HaloTag fusions after labeling with HTL-TMR (red) was performed as described for Fig. 5. Scale bars, 10 and 5 μm in overviews and details, respectively. Download FIG S6, JPG file, 2.6 MB. Copyright © 2019 Göser et al. 2019 Göser et al. This content is distributed under the terms of the Creative Commons Attribution 4.0 International license.

**FIG 6:**
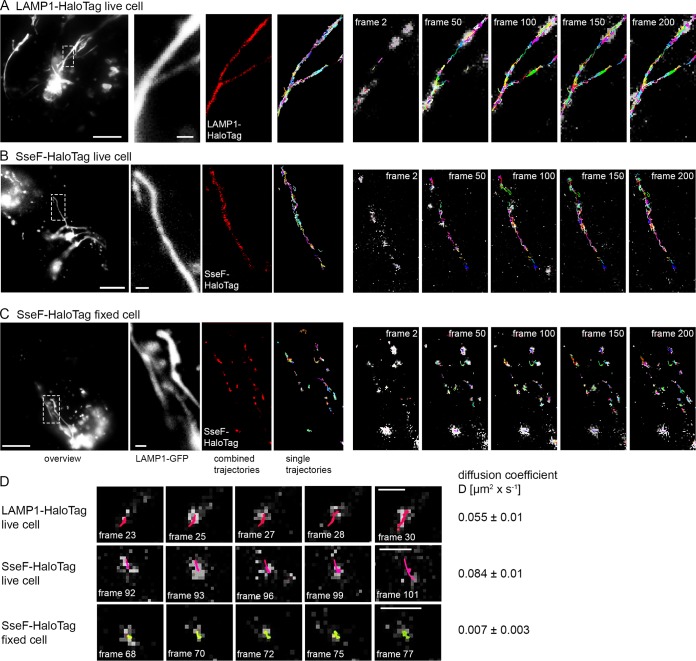
Single-molecule tracking of SPI2-T3SS effector protein SseF-HaloTag and host protein LAMP1-HaloTag. (A) HeLa cells stably expressing LAMP1-meGFP were transiently transfected for expression of LAMP1::HaloTag and infected with WT STM at an MOI of 75. (B) HeLa cells stably expressing LAMP1-meGFP were infected with the STM Δ*sseF* strain expressing *sseF*::HaloTag::HA at an MOI of 75. (C) Infection was performed as for panel B, but cells were fixed. After incubation for 7 h, labeling reactions were performed directly before imaging by HTL-TMR (20 nM final concentration) for 15 min at 37°C. Live-cell imaging was performed using 15% laser power at the focal plane, and single molecule localizations, and trackings within 200 consecutive frames were analyzed. Selected frames (frame rate: 32 frames per second) of HTL-TMR signals and localization and tracking (also showing elapsed trajectories) are presented. Each trajectory has a different color. Scale bars, 10 μm (overview), 1 μm (detail). The sequences of 200 frames show elapsed trajectories, and corresponding accumulated trajectories are shown in Movie S2 for LAMP1-HaloTag, SseF-HaloTag (live), and SseF-HaloTag (fixed), respectively. (D) Single-molecule tracks of singular trajectories from SseF-HaloTag and LAMP1-HaloTag were recorded in living cells over time. As a control, the diffusion of SseF-Halo-tag was monitored in fixed cells. Using at least 1,000 pooled trajectories of proteins from SIF structures in five infected cells recorded under the same conditions, the diffusion coefficient *D* was calculated using the Jaqaman algorithm.

We also generated strains with SLE fusions to effector genes in the native chromosomal localization. For this, a modified Red recombineering approach was used that allows the generation of translational gene fusions (15). The labeling and subcellular distribution of SseJ-HaloTag, PipB2-HaloTag, and SifA-HaloTag were indistinguishable between plasmid-borne and chromosomal effector-SLE fusion proteins (Fig. 6). We performed dual labeling of SseF-HaloTag-HA by SLE reaction with HTL-TMR and subsequent immunolabeling of HA tag with Cy5-conjugated antibody. SRM for TMR and Cy5 revealed a high degree of colocalization of the two signals (Fig. S7). This indicates that the majority of translocated SLE fusion proteins are capable of catalyzing the self-labeling reaction.

10.1128/mBio.00769-19.8 FIG S7 Dual labeling SRM of SseF-HaloTag-HA with SLE ligand HTL-TMR and antibody against HA tag. HeLa cells stably expressing LAMP1-meGFP (white in overlay) were infected with the STM *sseF* strain expressing *sseF*::HaloTag::HA at an MOI of 75. Incubation, labeling with HTL-TMR (red), and fixation were performed as described for Fig. 5. Additional immunolabeling was performed using primary antibody directed against HA (1:1,000) and secondary antibody against rat coupled to Cy5 (1:10,000) (blue). Dual-channel dSTORM imaging was performed as for Fig. 5. Representative SRM images rendered from single-molecule localizations within 500 consecutive frames are shown. Scale bars, 10 and 2.5 μm in overview and details, respectively. Download FIG S7, TIF file, 2.7 MB. Copyright © 2019 Göser et al. 2019 Göser et al. This content is distributed under the terms of the Creative Commons Attribution 4.0 International license.

10.1128/mBio.00769-19.10 MOVIE S2 Single-molecule tracking of LAMP1-HaloTag or SseF-HaloTag labeled with HTL-TMR in a STM-infected cell with SIF formation. The movie corresponds to the still images shown in Fig. 6A to C consisting of 200 frames per sequence. Single trajectories are shown in the right panel; the left panel shows accumulated trajectories. Scale bar, 1 μm. Download Movie S2, MPG file, 11.3 MB. Copyright © 2019 Göser et al. 2019 Göser et al. This content is distributed under the terms of the Creative Commons Attribution 4.0 International license.

In diffraction-limited analyses of translocated SPI2-T3SS effector proteins, a continuous distribution of effector proteins on *Salmonella*-containing vacuole and SIF membranes was frequently observed. SRM analyses indicate that the effector distribution is rather discontinuous, and patches of SIF membranes with high density of labeling as well as patches without effector were detected. This observation prompted us to investigate the dynamics of effector distribution in more detail.

### Single-molecule localization and tracking of translocated effector proteins.

Finally, we investigated the use of SLE fusion proteins to analyze the dynamics of effector proteins in living cells. SLEs labeled with fluorochrome-conjugated substrates have been used for single-molecule tracking approaches, and we tested if such analyses would be applicable to effector proteins in STM-infected cells. HeLa cells expressing LAMP1-GFP were infected with STM translocating SseF-HaloTag. HTL-TMR labeling was performed in living cells and live-cell imaging was performed recording 750 consecutive frames. In order to compare the dynamics of translocated effector proteins to those of a host cell protein with known membrane integral localization, we analyzed LAMP1-HaloTag dynamics in STM-infected HeLa cells (Fig. 6 and Movie S2), and diffusion coefficients *D* were calculated for at least 1,000 recorded trajectories. We determined *D* values of 0.055 (±0.01) μm^2^ · s^−1^ for host cell protein LAMP1. Translocated effector SseF revealed *D* values of 0.084 (±0.01) μm^2^ · s^−1^ and 0.007 (±0.003) μm^2^ · s^−1^ in living and fixed host cells, respectively. Both LAMP1 and SseF showed bidirectional motility along tubular membrane structures. The signals were confined to SIF tubules, and accumulated trajectories delineated the volume of the tubules. We observed similar properties in analyses with other SPI2-T3SS effector proteins fused to HaloTag.

These data show that SLE fusion proteins are applicable to analyses of single-molecule localization and dynamics in living host cells infected by a bacterial pathogen.

## DISCUSSION

**FIG 7:**
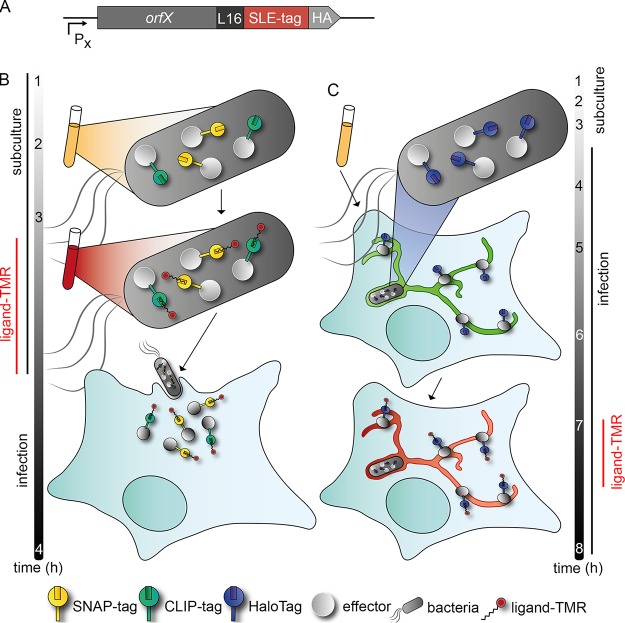
Principles of analyses of effector protein-SLE fusion proteins in infection biology. (A) Genetic fusions between T3SS effector proteins and SLE; for example, HaloTag is expressed by the bacterial pathogen. Fusion proteins are translocated and assume specific subcellular localization in host cells. Ligands are cell-permeable and allow labeling in living infected host cells or within bacteria. After removal of nonbound ligand, labeled effector proteins can be detected by various imaging approaches. (B) Labeling of effector-SLE fusion proteins with ligands in extracellular bacterial cells and translocation into host cells. This approach is applicable to preformed proteins such as SPI1-T3SS effector proteins. (C) Labeling of SLE fusion proteins in infected cells. This approach is applicable to proteins synthesized by intracellular bacteria, such as SPI2-T3SS effector proteins. Representative experimental time lines are given for analyses performed with STM SPI1-T3SS or SPI2-T3SS effector proteins.

Here we describe a novel approach for biorthogonal labeling of effector proteins of bacterial type III secretion systems using SLEs as tags. We demonstrated that effector-SLE fusion proteins are translocated by invading or intracellular bacteria, and that the presence of the SLE tags is compatible with the specific function of the effector protein after translocation into host cells. The basic concept of the approach is depicted in Fig. 7, and this technique now allows the application of the entire spectrum of SLE-based approaches to study bacterial effector proteins. This includes SRM for precise subcellular localization and TALM. To our knowledge, this is the first analysis of dynamics of effector proteins translocated by bacteria in living host cells. We have previously demonstrated the use of SLEs for analyses of bacterial secretion systems in bacterial cells (9, 14) and as universal markers for correlative light and electron microscopy (16). This methodology may also be applied to translocated effector proteins. Further applications include the selective capture of effector proteins by covalent binding to SLE ligand matrix for enrichment of effector proteins and host cell targets from infected cells.

SRM has been recently applied to the study of the T3SS translocon assembly during infection by Y. enterocolitica (17) and to analysis of the distribution of SPI2-T3SS effector protein SseJ in STM-infected cells (18). Due to the requirement for immunostaining, these analyses were restricted to fixed cells. Live-cell imaging of translocated effector proteins is a technical challenge, and only a few approaches have been devised to address this issue. Fusions to fluorescent proteins such as GFP would be an ideal experimental tool. However, the formation of a highly stable β-barrel structure of these proteins blocks transport by the T3SS and likely most other protein secretion systems in Gram-negative bacteria. Indeed, an N-terminal GFP moiety has been used to block translocation by the SPI1-T3SS and allowed arrest of the system with a transport intermediate of an effector protein (19). SLE fusion proteins are compatible with T3SS translocation. Interestingly, our data suggest that translocation of the ligand-bound form of SNAP-tag is possible, since prelabeled SopE-SNAP-tag was detected in infected host cells (Fig. 4). This could imply that an enzymatically active SLE is formed in the bacterial cytosol, catalyzes the self-labeling reaction, and then can assume a translocation compatible conformation. The SNAP-tag substrate benzylguanine-TMR (SNAP-Cell TMR-Star) is rather small, with a molecular weight of 677.1 Da.

While GFP and related fluorescent proteins cannot be used for analyses of effector translocation, bimolecular fluorescence complementation approaches turned out to be applicable to study effector translocation. A small peptide of GFP (GFP β-strand 11) is fused to the effector of interest. Upon translocation and interaction with nonfluorescent GFP β-strands 1 to 10, bimolecular fluorescence complementation is initiated. This approach allowed analyses of several SPI2-T3SS effector proteins (3, 20). Limitations of this approach are the slow maturation kinetics of split GFP and the requirement for host cells expressing the GFP β-strand 1 to 10 moiety.

The earliest approach for direct analysis of effector translocation was the use of the small tetracysteine tag and labeling with the biarsenic dye conjugate FlAsH (4). This approach allowed analyses of translocation of T3SS effector proteins during host cell invasion by Shigella flexneri (5) and SPI1-T3SS effector proteins SopE and SptP (21). The method is also applicable for SRM, as FlAsH-PALM has been used to monitor HIV in infected cells (22). This approach was applied to preformed and prelabeled effector proteins, and an application to effector proteins newly synthesized by intracellular bacteria is pending. As the dye shows toxic properties toward eukaryotic cells, effector labeling in a late stage of infection is problematic (23).

A recently described approach deploys the phiLOV domain, which endogenously binds chromophore flavin mononucleotide, yielding fluorescent properties. The delivery of preformed fusion proteins with Escherichia coli effector Tir and Shigella flexneri effector IpaB (24) into host cells was followed, allowing quantification analyses of translocation kinetics and amounts of effector proteins. Tagging and tracking of STM SPI1-T3SS effector SipA (25) and SPI2-T3SS effector SifA (26) were described. SRM and TALM approaches may not be possible due to selective binding of phiLOV to flavin mononucleotide.

A recent study exploited the SunTag system (27) to visualize the translocation of Shigella flexneri effector IcsB (28). This novel system uses a protein scaffold, a repeating peptide array termed SunTag, which is able to recruit multiple copies of an antibody-fusion protein. Translocation of a SunTag fused effector in host cells expressing eGFP-fused single-chain anti-SunTag antibody led to antibody recruitment and thus a fluorescence increase (28).

The SLE-based approach described here allows expression of genes for fusion proteins under the control of the native promoters, either on low-copy-number vectors or in their chromosomal context. In contrast, the split GFP approach yields rather low fluorescence intensities, demanding use of stronger promoters (20).

The current limitations of SLE fusion to effector proteins should be considered. Although compatible with translocation by the T3SS, the presence of SLE moiety is likely to affect the efficiency of translocation and may alter subcellular localization and interfere with effector function. While our analyses did not indicate the last artifact, careful controls will be required for application to other effector proteins.

We observed strong labeling of effectors fused to SLE tags inside bacterial cells. This might point to incomplete translocation of the total effector pool. Further work is required to determine the proportion of effector molecules translocated versus retained in the producing bacterial cell. If only a fraction of the effector pool is translocation compatible, the design of the fusion constructs may be altered by changing the intramolecular position of the SLE tags, altering linker sequences, or designing minimal SLE tags.

The choice of SLE appears to be of critical importance. We found that HaloTag fusions are functional for effector proteins of the SPI2-T3SS but not for those of the SPI1-T3SS or the *Yersinia* T3SS. The opposite was observed with SNAP-tag and CLIP-tag. While SPI2-T3SS effector proteins are synthesized *de novo* during the intracellular presence of STM in host cells, SPI1-T3SS and *Yersinia* effector proteins are preformed and delivered upon contact to the host cells. The kinetics of synthesis and translocation may explain the distinct applicability of the SLE for certain effector proteins. A further parameter may be differences in the inner diameter of the T3SS needle and/or translocator structure and the mode of loading of effector proteins into the T3SS. While the molecular basis of this observation needs further investigation, we recommend an evaluation of different SLEs regarding their performance for applications to new effector proteins. While STM and *Yersinia* have been used for validation in this work, application to other pathogens deploying effector translocation via T3SS, or to other bacterial secretion systems such as T4SS or T6SS, should be possible. This again may require evaluation of the most suitable SLE tag for a given pathogen and secretion system.

The dye TMR used in this study, as well the ligands conjugated to SiR (silicon rhodamine), allow application of the stochastic SRM techniques resulting in highly increased spatial resolution, typically in the range of 20 to 25 nm. The resolution should allow the analyses of the precise subcellular localization of effector proteins in relation to the complex membrane organization of the host cell and with respect to defined host cell proteins.

Another field for future optimization of the approach is the design of SLE ligands. Here we used TMR as dye conjugated to distinct ligands for HaloTag, SNAP-tag, or CLIP-tag. TMR is a cell-permeable dye able to enter mammalian as well as bacterial cells. Despite the use of the same dye, the specific SLE ligands resulted in background labeling to different extents. While HTL-TMR showed only low background signals, the SNAP-tag ligand generated background too high to allow detection of specific signals. When working with the ligand for SNAP-tag in eukaryotic cells, it must be considered that a high background staining can affect imaging. Thus, improved ligand dye conjugates are desired. While variations of the ligand portion may be limited by the requirements of substrate specificity, the dye portion may allow a wider range of optimization. One example is the SiR dye (29). To allow application of live-cell imaging, SRM, and single-molecule tracking, blinking properties of the dye are prerequisite. Using the poststaining approach for effector proteins translocated into host cells, extensive washing was required to remove nonbound ligands. These washing steps may affect host cell functions and cause delays in imaging experiments with high temporal resolution. Ligands with increased fluorescence after binding to the SLE tags are desirable, whereby “no-wash” experiments may be possible. A compilation of current advantages and limitations of the SLE tag approach for translocation analysis of bacterial effector proteins is provided in Table 1.

**TABLE 1:** Advantages and limitations of SLE tags for analyses of effector protein translocation

Parameter	Advantage	Limitation
SLE tag	Fast formation of stable covalent bonds	High molecular mass (20–33 kDa)
Substrate	Cell permeable, nontoxic; various fluorescent dyes can be coupled	SNAP-tag substrates show high background staining in host cells.
Compatibility	Effectors of *Yersinia* and *Salmonella*, both SPI1-T3SS and SPI2-T3SS	Reduced translocation of fusion proteins, SLEs varying in applicability for different effector groups
Labeling	Pre- and postlabeling possible	Washing steps are required.
Application	Confocal microscopy, SRM, TALM *in vitro* and in cell-based models	No *in vivo* experiments
Expression	Native promoters can be used. Expression by low-copy-number vectors or by chromosomal genes	

The single-molecule tracking of SPI2-T3SS effector SseF showed a rapid movement comparable to the mobility of host transmembrane protein LAMP1 along the SIF tubule. The movement has a bidirectional pattern. Lateral movement of the STM effector PipB2 along tubules was already demonstrated with the split GFP method using fluorescence recovery after photobleaching (3). The availability of the TALM approach for infected host cells will enable in-depth analyses of the delivery mode of effector proteins, their fate inside host cells, and the mechanisms of trafficking to their proper subcellular localization. While host cell-mediated modification was shown to control the positioning of certain effector proteins (30), the molecular mechanisms of targeting most effector proteins to specific host cell compartments are still elusive. For example, this holds true for the SPI2-T3SS effector proteins that are associated with membranes of the tubular SIF network. We have recently performed in living host cells systematic analyses of the dynamics of various SPI2-T3SS effector proteins on distinct forms of SIF membranes, and we also used effector-SLE fusion proteins to follow the fate of effector proteins from time points early after translocation to the final destination in host cell endosomal membranes (V. Göser and M. Hensel, unpublished observations). Our approach allows novel forms for single-cell, single-molecule analyses in living infected cells and by this may open doors to the molecular understanding of bacterial pathogenesis.

## MATERIALS AND METHODS

### Bacterial strains and culture conditions.

For this study Salmonella enterica serovar Typhimurium (STM) NCTC12023 was used as a wild-type (WT) strain. All mutant strains are isogenic to WT, and Table 2 shows the characteristics of strains used in this study. STM strains were routinely grown on LB agar or in LB broth containing 50 μg · ml^−1^ of carbenicillin for maintenance of plasmids at 37°C using a roller drum. The Yersinia enterocolitica strain, WA-C, used in this study is a derivate of the serotype O:8 strain and was virulence plasmid cured (31). For effector translocation the strain harbors the plasmid pTTSS, encoding the TTSS apparatus (32). *Yersinia* strains were routinely grown on LB agar or in LB broth containing 100 μg · ml^−1^ of spectinomycin and 12.5 μg · ml^−1^ of chloramphenicol for maintenance of plasmids at 30°C using a roller drum.

**TABLE 2:** Bacterial strains used in this study

Organism	Relevant characteristics	Source or reference
Salmonella enterica serovar Typhimurium		
NCTC12023	Wild type	NCTC Colindale, lab stock
MvP392	Δ*sseJ*::FRT	44
MvP503	Δ*sifA*::FRT	45
MvP818	Δ*invC*::FRT	46
MvP1890	Δ*ssaV*::FRT	47
MvP1980	Δ*sseF*::FRT	47
MvP1944	Δ*pipB2*::FRT	38
MvP2346	*sseJ*::HaloTag::*aph*	This study
MvP2365	*sifA*::HaloTag::*aph*	This study
MvP2362	*pipB2*::HaloTag::*aph*	This study
M712	Δ*sipA* Δ*sopA* Δ*sopB* Δ*sopE* Δ*sopE*2	11
Yersinia enterocolitica WA-C, virulence plasmid cured		31

### Generation of plasmids and strains for expression of effector-SLE fusions.

Plasmids for synthesis of SLE-tagged effector proteins used in this study were constructed using Gibson Assembly and are listed in Table 3. Primers used for cloning and sequencing are listed in Table 4. DNA fragments encoding HaloTag, SNAP-tag, or CLIP-tag were amplified from vector p3780, p3779, or p4191, respectively, and inserted in already existing plasmids harboring effector proteins with an HA epitope tag. Behind sequences for effector proteins a L16 linker sequence was included (5′-GGCTCTGCGGCGTCTGCGGCGGGCGCGGGCGAAGCGGCGGCG-3′, encoding GSAASAAGAGEAAA). Alternatively, genes encoding effector proteins were amplified from genomic DNA of STM NCTC12023 and inserted and exchanged for effectors on preexisting plasmids harboring effectors with HaloTag, SNAP-tag, or CLIP-tag, as well as an HA epitope tag. Chromosomal fusions of *pipB2*, *sifA*, and *sseJ* with the HaloTag were constructed as described previously (15, 33). Briefly, gene cassettes encoding HaloTag and kanamycin resistance were amplified by PCR. After purification and DpnI digestion, the PCR product was electroporated into competent STM cells harboring pWRG730. The *aph* gene was removed using FLP-mediated recombination (34).

**TABLE 3:** Plasmids used in this study

Plasmid	Relevant genotype	Reference
p3779	Template vector for L16::SNAP-tag::HA	9
p3780	Template vector for L16::HaloTag::HA	9
p4191	Template vector for L16::CLIP-tag::HA	9
p2643	P*_sseA_ sscB sseF*::HA	48
p4118	P*_sseA_ sscB sseF*::L16::HaloTag::HA	This study
p4123	P*_sseA_ sscB sseF*::L16::SNAP-tag::HA	This study
p4192	P*_sseA_ sscB sseF*::L16::CLIP-tag::HA	This study
p3622	P*_sifA_ sifA*::M45	45
p4305	P*_sifA_ sifA*::L16::HaloTag::HA	This study
p4307	P*_sifA_ sifA*::L16::SNAP-tag::HA	This study
p4306	P*_sifA_ sifA*::L16::CLIP-tag::HA	This study
p2684	P*_sseJ_ sseJ*::HA	48
p4286	P*_ssej_ sseJ*::L16::HaloTag::HA	This study
p4287	P*_sseJ_ sseJ*::L16::SNAP-tag::HA	This study
p4285	P*_sseJ_ sseJ*::L16::CLIP-tag::HA	This study
p2621	P*_pipB2_ pipB2*::M45	49
p4295	P*_pipB2_ pipB2*::L16::HaloTag::HA	This study
p4291	P*_pipB2_ pipB2*::L16::SNAP-tag::HA	This study
p4293	P*_pipB2_ pipB2*::L16::CLIP-tag::HA	This study
p4040	P*_sipA_ sipA*::HA	50
p4115	P*_sipA_ sipA*::L16::HaloTag::HA	This study
p4120	P*_sipA_ sipA*::L16::SNAP-tag::HA	This study
p4043	P*_sopE_ sopE*::HA	50
p4117	P*_sopE_ sopE*::L16::HaloTag::HA	This study
p4122	P*_sopE_ sopE*::L16::SNAP-tag::HA	This study
p4196	P*_sopE_ sopE*::L16::CLIP-tag::HA	This study
p4042	P*_siopB_ sopB*::HA	50
p4116	P*_sopB_ sopB*::L16::HaloTag::HA	This study
p4121	P*_sopB_ sopB*::L16::SNAP-tag::HA	This study
p4195	P*_sopB_ sopB*::L16::CLIP-tag::HA	This study
pTTSS	Y. enterocolitica T3SS	32
pYopM	Effector gene *yopM*	32
p4798	P*_yopM_ yopM*::L16::HaloTag::HA	This study
p4926	P*_yopM_ yopM*::L16::CLIP-tag::HA	This study
p4927	P*_yopM_ yopM*::L16::SNAP-tag::HA	This study
pWRG730	Red recombinase expression	33
pE-FLP	FLP recombinase expression	51

**TABLE 4:** Oligonucleotides used in this study

Purpose and designation	Sequence (5′–3′)
Cloning	
Vr-pWSK 29-2	GGTACCCAATTCGCCCTATAGTGAGTCGTATTAC
Vf-16-2	GGCTCTGCGGCGTCTGCGGCGGGC
Vf-L16	GGCTCTGCGGCGTCTGCG
Vr-p2643	TGGTTCTCCCCGAGATGT
Vr effector HA	TTAAGCGTAGTCTGGGACGTCGTATGGGTACTCC
1f-sseF-L16	ACATCTCGGGGAGAACCAGGCTCTGCGGCGTCTGCG
1f-sifA	TAGGGCGAATTGGGTACCAGGTGAGTGATATAAGCGATTAATTGC
1r-sifA	CGCAGACGCCGCAGAGCCTAAAAAACAACATAAACAGCCGCTTT
1f-sseJ	TAGGGCGAATTGGGTACCTTAATATGAAAATAGAAATCAAAATGTCACATAA
1r-sseJ	CGCAGACGCCGCAGAGCCTTCAGTG-GAATAATGATGAGCTA
1f-pipB2	TAGGGCGAATTGGGTACCTAATAAAATGCCTGAACACG
1r-pipB2	CGCAGACGCCGCAGAGCCAATATTTTCACTATAAAATTCGTTAAAG
1f-sipA-L16	GGCTTGCACATGCAGCGTGGCTCTGCGGCGTCTGCG
1f-sopB-L16	AGGCATTTCTTCATTAATCACATCTGGCTCTGCGGCGTCTGCG
Vr-p4040	ACGCTGCATGTGCAAGCC
Vr-p4042	AGATGTGATTAATGAAGAAATGCCTTT
Vr-p4043	GGGAGTGTTTTGTATATATTTATTAGCAATGT
1r-SNAP_CLIP-HA	TGGGACGTCGTATGGGTAACCCAGCCCAGGCTTGCC
Vf-HA	TACCCATACGACGTCCCAGA
Sequencing	
HaloTag-Check-Rev	TGGAAAGCCAGTACCGATTT
SnapTag-Check-Rev	CTTCATTTCGCAGTCTTTGT
Seq-For	CGCCAGGGTTTTCCCAGTCACGAC

### Cell culture.

The murine macrophage line RAW264.7 (American Type Culture Collection; ATCC no. TIB-71) was cultured in high-glucose (4.5 g · ml^−1^) Dulbecco’s modified Eagle’s medium (DMEM) containing 4 mM stable glutamine (Merck) and supplemented with 6% inactivated fetal calf serum (Sigma). The nonpolarized epithelial cell line HeLa (ATCC no. CCL-2) was cultured in high-glucose DMEM containing 4 mM stable glutamine and sodium pyruvate and supplemented with 10% inactivated fetal calf serum. Stably transfected HeLa cell lines expressing LAMP1-GFP or LifeAct-GFP were cultured under the same conditions. All cells were maintained at 37°C in an atmosphere containing 5% CO_2_ and absolute humidity.

### Detection of secreted effector proteins by Western blotting.

STM strains cultured overnight were subcultured (1:31) in LB broth for 6 h at 37°C. *Yersinia* strains cultured overnight at 30°C were subcultured (1:150) in LB broth at 37°C for 90 min. Secretion of effectors was induced as described previously (35). In short, 15 mM MgCl_2_, 5 mM EGTA, and 0.2% glucose were added to the culture and subcultured for additional 2 h. The supernatant was precipitated overnight at 4°C with 20% trichloroacetic acid. The pellets of precipitated supernatants were washed twice with acetone and air dried. Total bacterial cells and precipitated secreted proteins were dissolved in SDS-PAGE loading buffer containing 0.1% glycine-HCl, pH 2.2, and boiled for 5 min. Ten-microliter volumes of samples were loaded onto a 12% SDS-PAGE gel. After electrophoresis, samples were blotted onto a 0.22-μm nitrocellulose membrane using a semidry electrophoretic transfer unit (Bio-Rad). Blots were incubated with a primary antibody directed against the HA epitope tag (1:1,000) and a secondary antibody anti-rat IgG antibody conjugated to horseradish peroxidase (HRP). Secondary antibody was detected by the ECL detection kit (Pierce), and blots were visualized with a Chemidoc imaging system (Bio-Rad).

### Gentamicin protection assay.

The gentamicin protection assay was performed as described by others (36). Briefly, RAW264.7 cells were seeded 24 or 48 h prior to infection in a surface-treated 24-well plate (TPP) to reach confluency (∼4 × 10^5^ cells per well) on the day of infection. Bacteria were grown overnight (∼20 h) aerobically in LB medium, adjusted to an optical density at 600 nm (OD_600_) of 0.2 in phosphate-buffered saline (PBS), and further diluted in DMEM for infection of RAW264.7 cells at a multiplicity of infection (MOI) of 1. Bacteria were centrifuged onto the cells for 5 min at 500 × *g*, and the infection was allowed to proceed for 25 min. After three washing steps with PBS, medium containing 100 μg · ml^−1^ of gentamicin was added for 1 h to kill extracellular bacteria. Afterwards the cells were incubated with medium containing 10 μg · ml^−1^ of gentamicin for the ongoing experiment. Cells were washed three times with PBS and lysed using 0.1% Triton X-100 at 2 and 16 h postinfection. CFU were determined by plating serial dilutions of lysates and inoculum on Mueller-Hinton II (MH) agar and incubated overnight at 37°C. The percentage of phagocytosed bacteria as well as the replication rate was calculated.

### Invasion assay.

HeLa cells were seeded in surface-treated 24-well plates (TPP) 24 or 48 h prior to infection to reach confluency (∼2 × 10^5^). Bacteria were subcultured from an overnight culture (1:31) in fresh LB medium and grown for 3.5 h at 37°C. The infection was done as described above at an MOI of 1. The inoculum and the lysates at 1 h postinfection were plated in serial dilutions onto MH agar. The percentage of internalized bacteria was calculated.

### Infection experiments for microscopy.

HeLa cells stably transfected and expressing either LAMP1-GFP or LifeAct-GFP were seeded in 24-well plates (TPP) on coverslips, 6-well plates (TPP) on coverslips, or 8-well (ibidi) plates. The cells were allowed to grow to 80% confluency (24-well plates, ∼1.8 × 10^5^; 8-well plates, ∼8 × 10^4^; and 6-well plates, ∼6 × 10^5^) on the day of infection. The cells were infected with STM strains as described above with 3.5-h bacterial subcultures. For SPI1-T3SS effector protein detection, an MOI of 100 was used and the cells were infected for 25 min. For SPI2-T3SS effector protein detection, an MOI of 50 was used and cells were infected for 8 h or 16 h. Next, the cells were either imaged directly or washed three times with PBS and fixed with 3% paraformaldehyde (PFA) in PBS. *Yersinia* strains were cultured overnight at 30°C, inoculated 1:150 in fresh medium, and subcultured for 90 min at 37°C with agitation in a roller drum. Bacteria were adjusted to an OD_600_ of 0.2 in PBS and further diluted in DMEM for infection at an MOI of 100. After incubation for 45 min, the cells were washed three times with PBS and directly fixed with 3% PFA.

### Immunostaining and fluorescence microscopy.

Immunostaining was performed as described by Müller et al. (37). Briefly, PFA-fixed cells were washed three times with PBS and incubated in blocking solution (2% goat serum, 2% bovine serum albumin [BSA], and 0.1% saponin in PBS) for 30 min. Afterwards cells were stained for 1 h at room temperature with the primary antibodies anti-HA (1:500), anti-M45 (1:10), anti-*Salmonella* O (1:500), and anti-*Yersinia* O (1:500). Secondary antibodies were selected accordingly and samples were incubated for 1 h (Table 5). Coverslips were mounted with Fluoroprep (bioMérieux) and sealed with Entellan (Merck). The microscopy of fixed samples was performed with a Leica SP5 confocal laser-scanning microscope using the 100× objective (HCX PL APO CS 100×; numerical aperture [NA], 1.4 to 0.7) and the polychroic mirror TD 488/543/633 for the three channels GFP, TMR/Alexa Fluor 568, and Cy5 (Leica, Wetzlar, Germany). For image processing, LAS-AF software (Leica, Wetzlar, Germany) was used.

**TABLE 5:** Antibodies and antisera used in this study

Antibody or antiserum	Characteristics	Reference or source
Anti-M45	Mouse anti-M45 epitope tag	52
Anti-HA	Rat anti HA epitope tag	Roche
*Salmonella* O antigen	Rabbit anti-*Salmonella* O antiserum	BD Difco
	Group B factors 1, 4, 5, 12	
*Yersinia* O-Ag	Rabbit anti-*Yersinia* O8 antiserum	Sifin
Anti-DnaK	Mouse anti-DnaK	Stressgen
Anti-mouse IgG Alexa Fluor 568	Goat anti-mouse IgG Alexa Fluor 568	Thermo Fisher
Anti-rat IgG Alexa Fluor 568	Goat anti-rat IgG Alexa Fluor 568	Thermo Fisher
Anti-rabbit IgG Cy5	Goat anti-rabbit IgG Cy5	Jackson ImmunoResearch
Anti-rat IgG Cy5	Goat anti-rat IgG Cy5	Jackson ImmunoResearch

### Labeling of SLE tags with TMR ligands.

The self-labeling of SPI2-T3SS effector proteins fused with HaloTag, SNAP-tag, and CLIP-tag was done with HaloTag ligand TMR (HTL-TMR; Promega), SNAP-tag ligand TMR (SNAP-Cell TMR-Star; New England BioLabs [NEB]), and CLIP-tag ligand TMR (CLIP-Cell TMR-Star; NEB) (excitation wavelength, 545 nm; emission wavelength, 575 nm). For live-cell fluorescence microscopy, HeLa LAMP1-GFP cells were infected for 16 h in 8-well plates. The cells were stained with appropriate substrate at a 1 μM final concentration for 30 min at 37°C. Afterwards, cells were washed three times with PBS and directly imaged. For superresolution microscopy, labeling reactions were done directly before fixation. HeLa LAMP1-GFP cells were seeded in a 6-well plate on coverslips and infected for 8 h. The cells were stained with 20 nM TMR-HTL for 15 min at 37°C and washed 10 times with DMEM. For single-molecule tracking, cells were labeled under the same conditions and live-cell imaging was performed directly after labeling reactions. The self-labeling of STM SPI1 effectors, or *Yersinia* effectors fused to HaloTag, SNAP-tag, or CLIP-tag, was done with the appropriate substrate. Either bacterial cells were prestained in the 3.5-h subculture used for infection experiments with 1 μM substrate for 30 min or the labeling reaction was done during the infection for 25 min of HeLa LifeAct-GFP or HeLa LAMP1-GFP cells by adding 1 μM substrate directly to the cell culture media.

### Live-cell microscopy.

Live-cell imaging was performed as described elsewhere (38). In short, the Leica SP5 was used with the settings described above. Additionally, the incubation chamber maintaining 37°C and humidity was activated. For imaging, DMEM was replaced with imaging medium consisting of minimal essential medium (MEM) with Earle’s salts and without NaHCO_3_, l-glutamine, or phenol red (Biochrom) and supplemented with 30 mM HEPES (Sigma-Aldrich), pH 7.4.

### Superresolution microscopy and localization and tracking microscopy.

For dSTORM experiments, total internal-reflection fluorescence (TIRF) microscopy of fixed cells was performed using an inverted microscope (Olympus IX-71) equipped with a motorized 4-line TIRF condenser (Olympus), a 150× objective (UAPON 150× TIRF; NA, 1.45), and high-power lasers: 488 nm and 200 mW (LuxX 488-200; Omicron) and 561 nm and 150 mW (Cobolt Jive 561; Cobolt). Images were acquired by an electron-multiplying charge-coupled-device (CCD) camera (Andor iXon Ultra 897), a quad-band polychroic mirror (zt405/488/561/640rpc; Semrock) for the two channels GFP and TMR, and 100% laser power (1.5 kW/cm^2^) for 561-nm illumination. For each cell, a snapshot for 488 nm and 500 frames for the 561-nm laser with an exposure time of 32 ms were recorded. Oxygen depletion was achieved by incubating the cells in a buffer containing 100 mM β-mercaptoethylamine, 4.5 mg · ml^−1^ of d-glucose, 40 μg · ml^−1^ of catalase, and 0.5 mg · ml^−1^ of glucose-oxidase. For dual-color dSTORM experiments with doubly labeled SseF-HaloTag-HA with the ligand TMR-HTL and immunostaining of the HA epitope tag additionally, a quad-band bandpass filter (FF01 446/523/600/677; Semrock) was used to filter the emitted light from the sample. Single-molecule localization and tracking microscopy (TALM) experiments with living cells were performed using an inverted microscope (Olympus IX-81) equipped with an incubation chamber maintaining 37°C and humidity, a motorized 4-line TIRF condenser (Olympus), a 150× objective (UAPON 150× TIRF; NA, 1.45), and a 488-nm laser (150 mW) as well as a 561-nm laser (150 mW). Cells were imaged 1 frame for the 488-nm laser and 150 frames for the 561-nm laser with an intensity of 5% and 15% laser power at the focal plane in a cycle of five repetitions with an exposure time of 32 ms. For imaging living cells, imaging medium was used as described above. TALM was performed as described in reference 39. Briefly, localization and tracking were carried out using a self-written user interface in Matlab 2013a (MathWorks). Localization is based on the multiple-target tracing algorithm established by Serge et al., and tracking was done with the help of the ultrack algorithm by Jaqaman et al. and subsequent adaptations (9, 40–43).
