# Supplementary figures and images for: Self-Labeling Enzyme Tags for Analyses of Translocation of Type III Secretion System Effector Proteins
Source: mBio. 2019 Jun 25;10(3):e00769-19. doi: 10.1128/mBio.00769-19 (PMC6593401; doi:10.1128/mBio.00769-19)

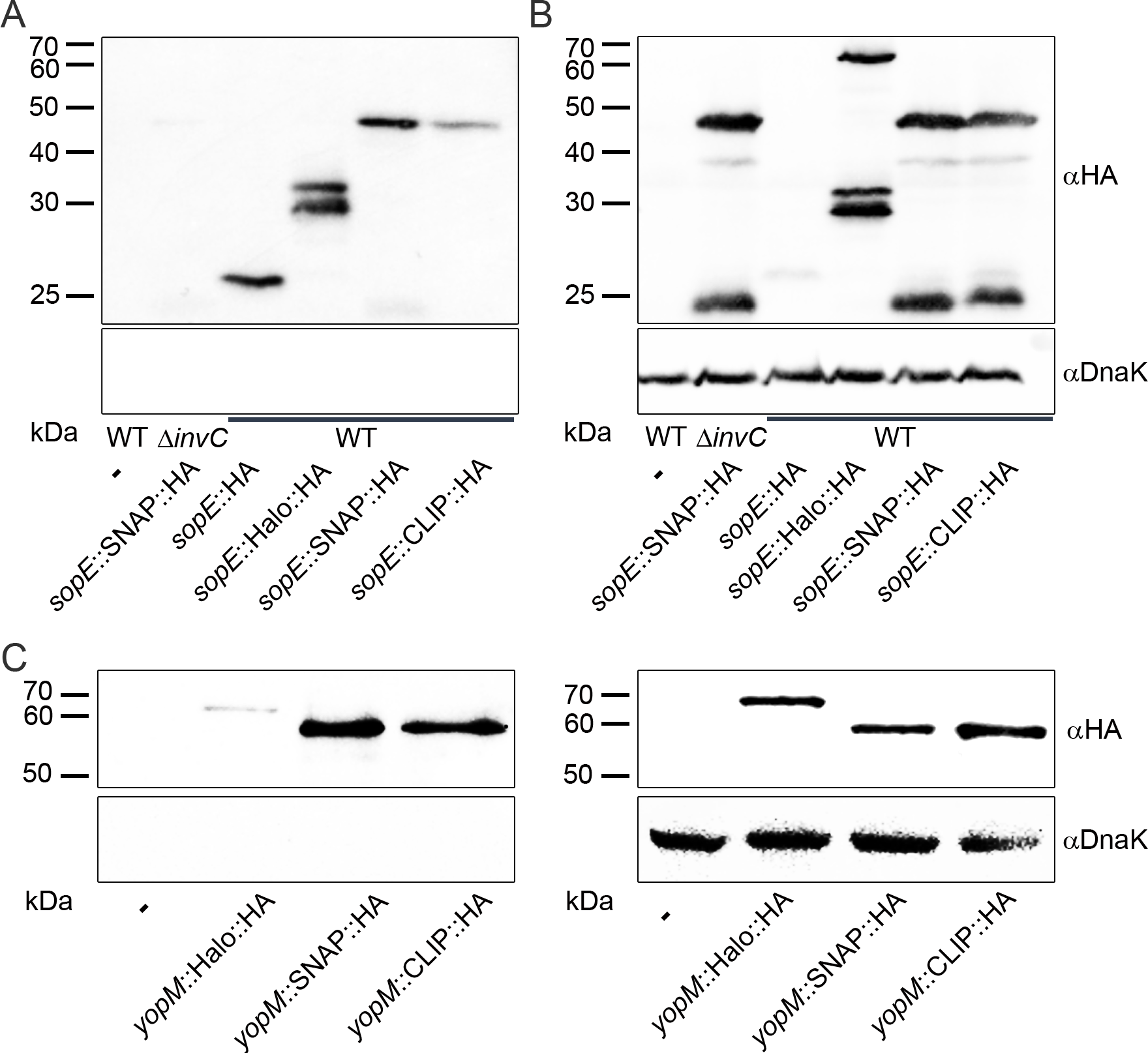

Supplement: FIG S1 [file mBio.00769-19-sf001.tif]

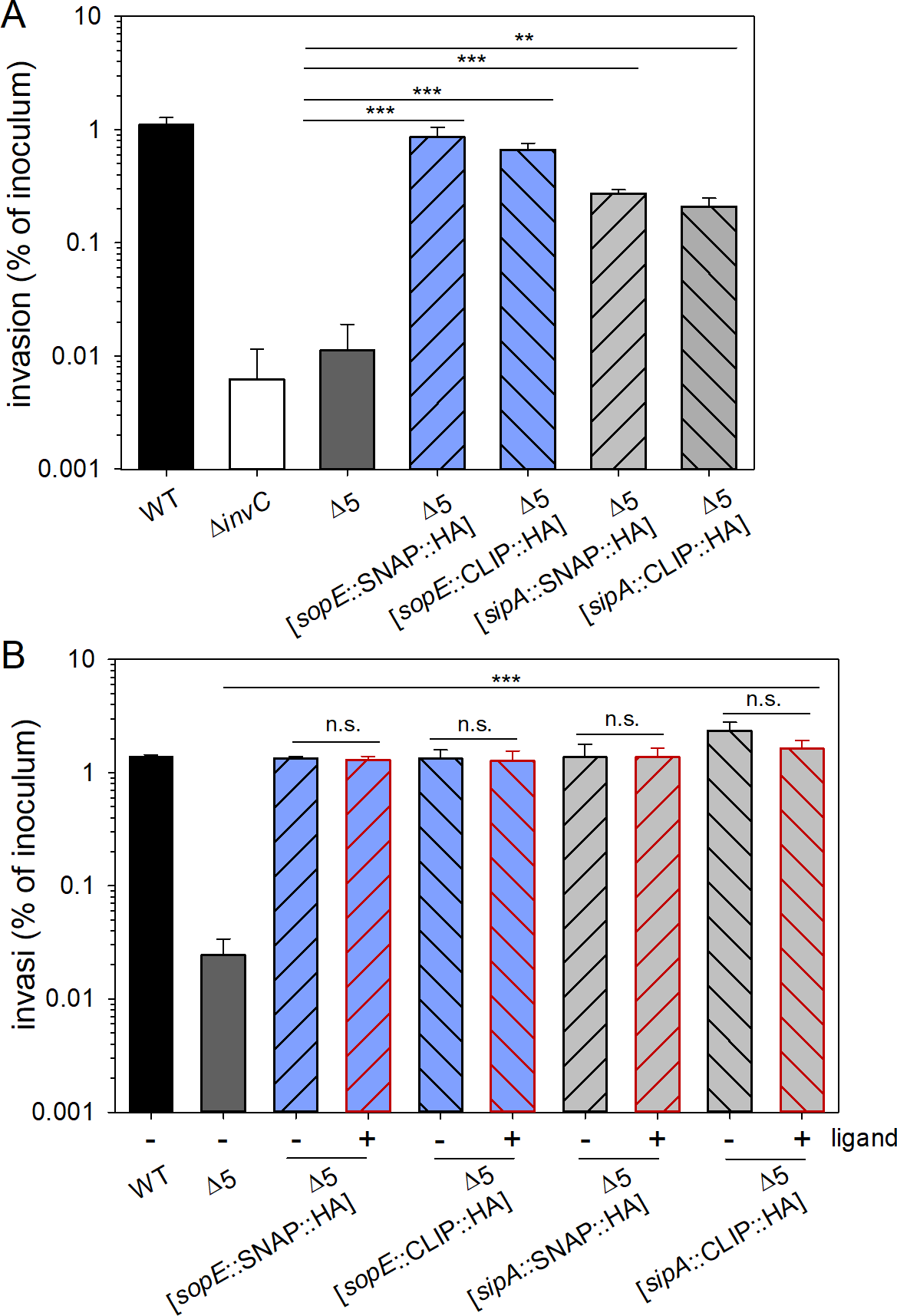

Supplement: FIG S2 [file mBio.00769-19-sf002.tif]

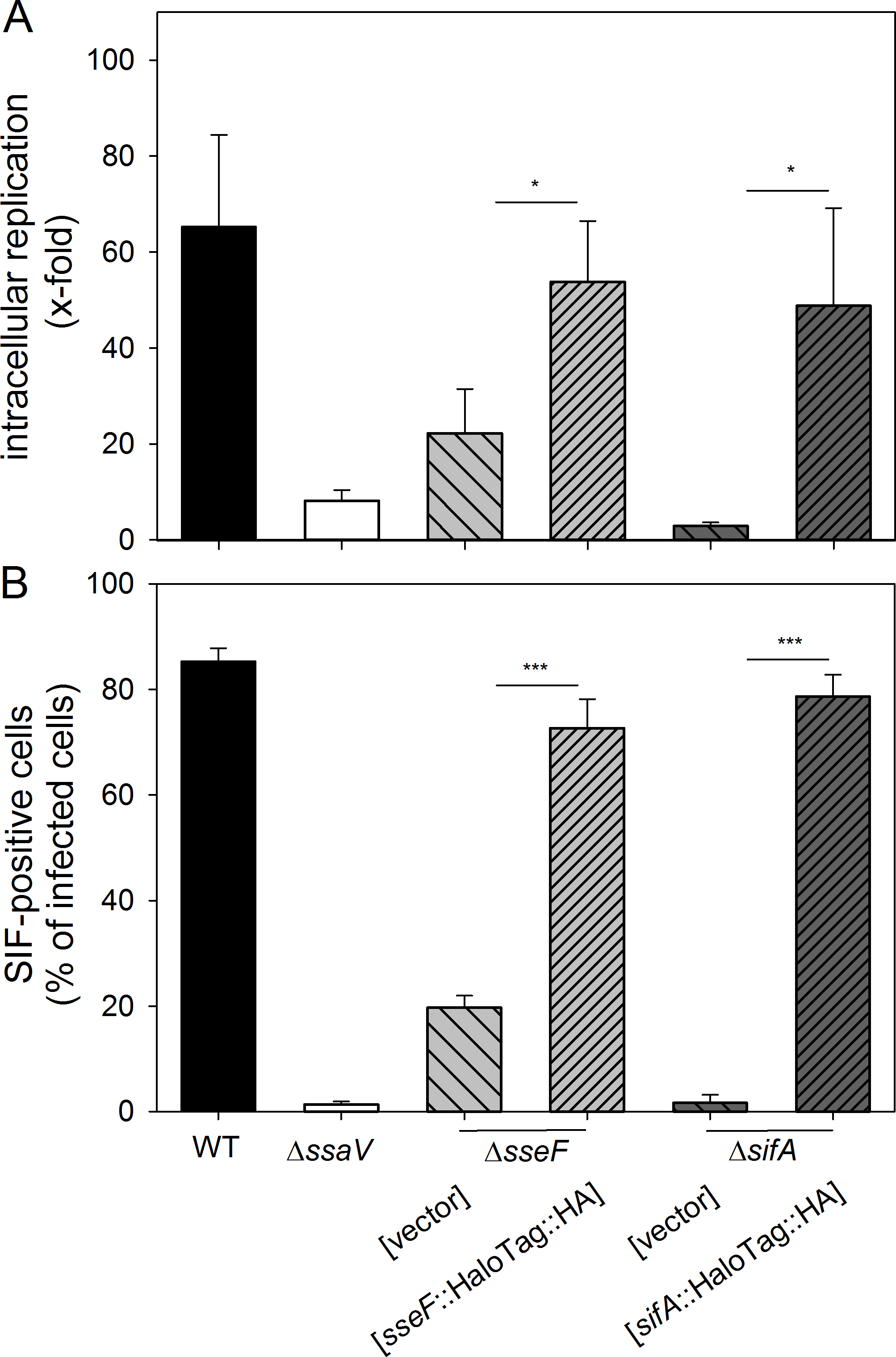

Supplement: FIG S3 [file mBio.00769-19-sf003.tif]

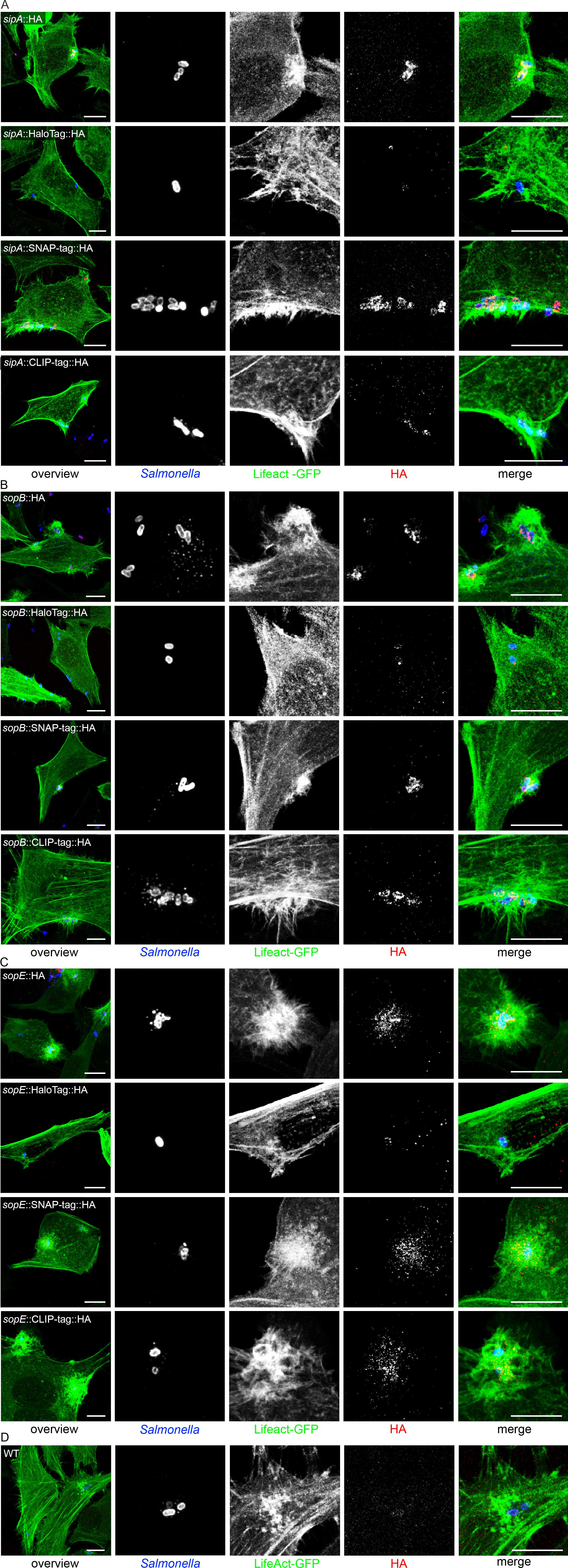

Supplement: FIG S4A [file mBio.00769-19-sf004.jpg]

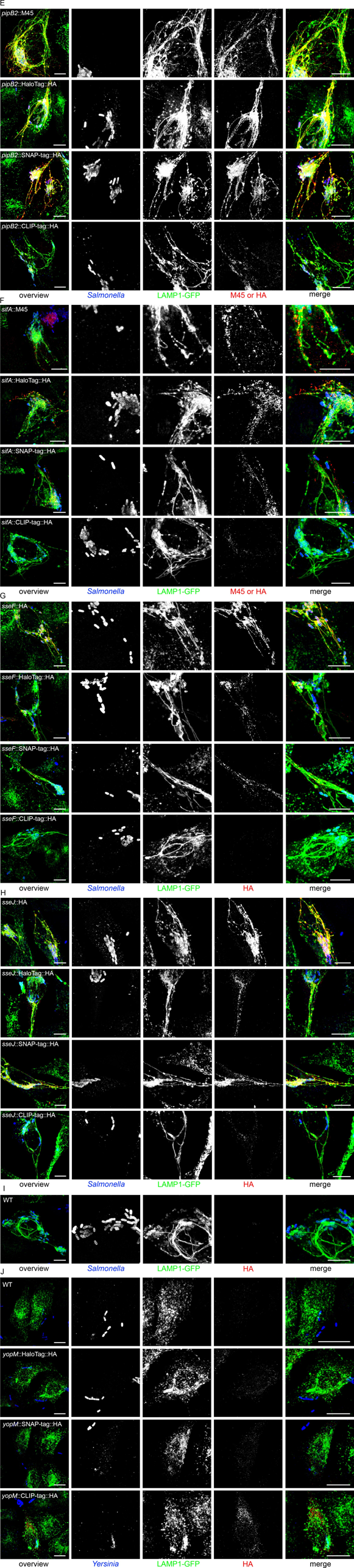

Supplement: FIG S4B [file mBio.00769-19-sf04e.jpg]

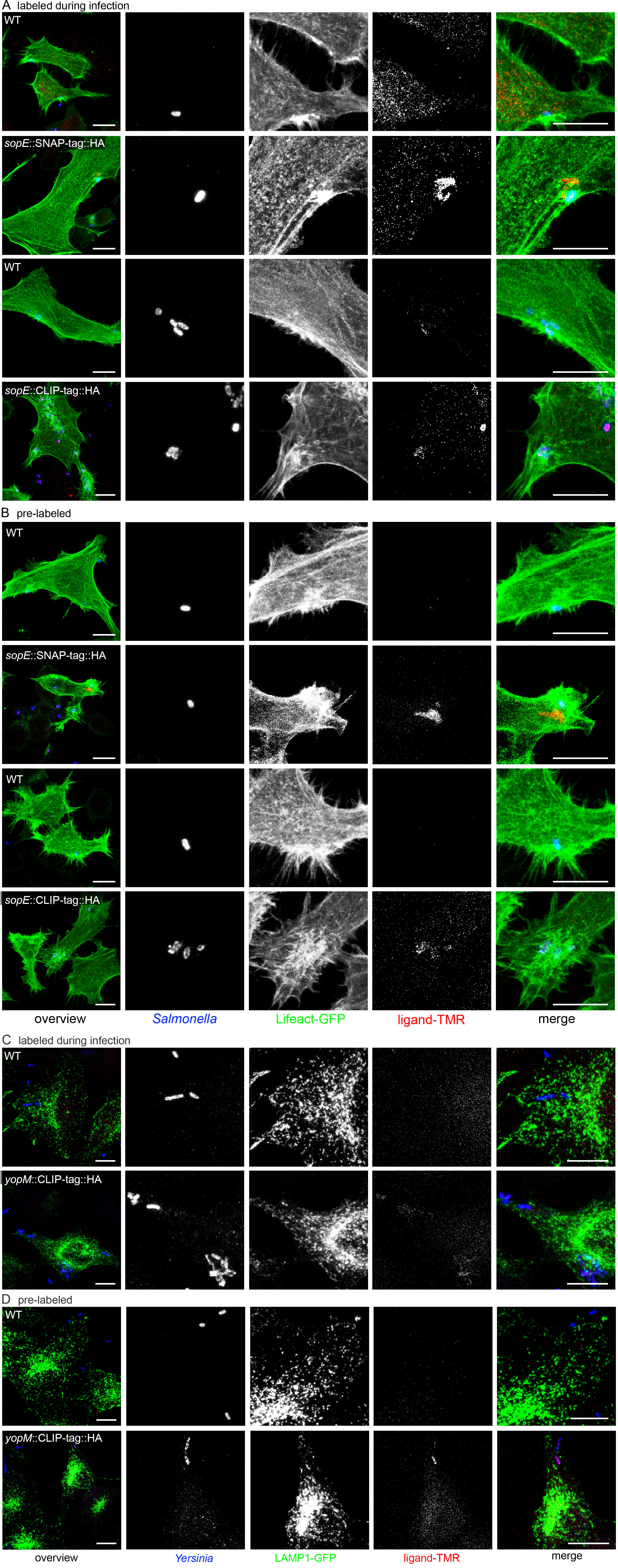

Supplement: FIG S5 [file mBio.00769-19-sf005.jpg]

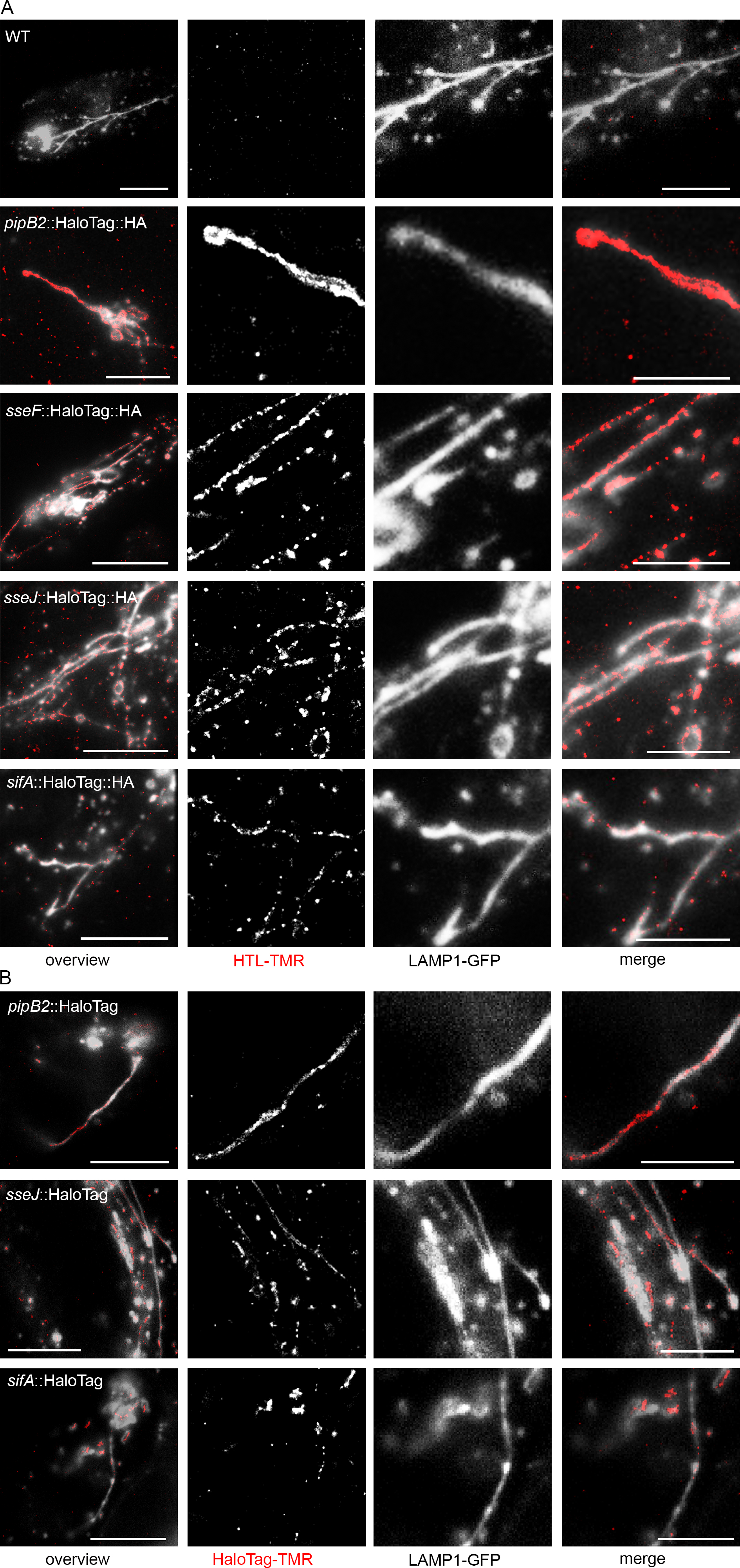

Supplement: FIG S6 [file mBio.00769-19-sf006.jpg]

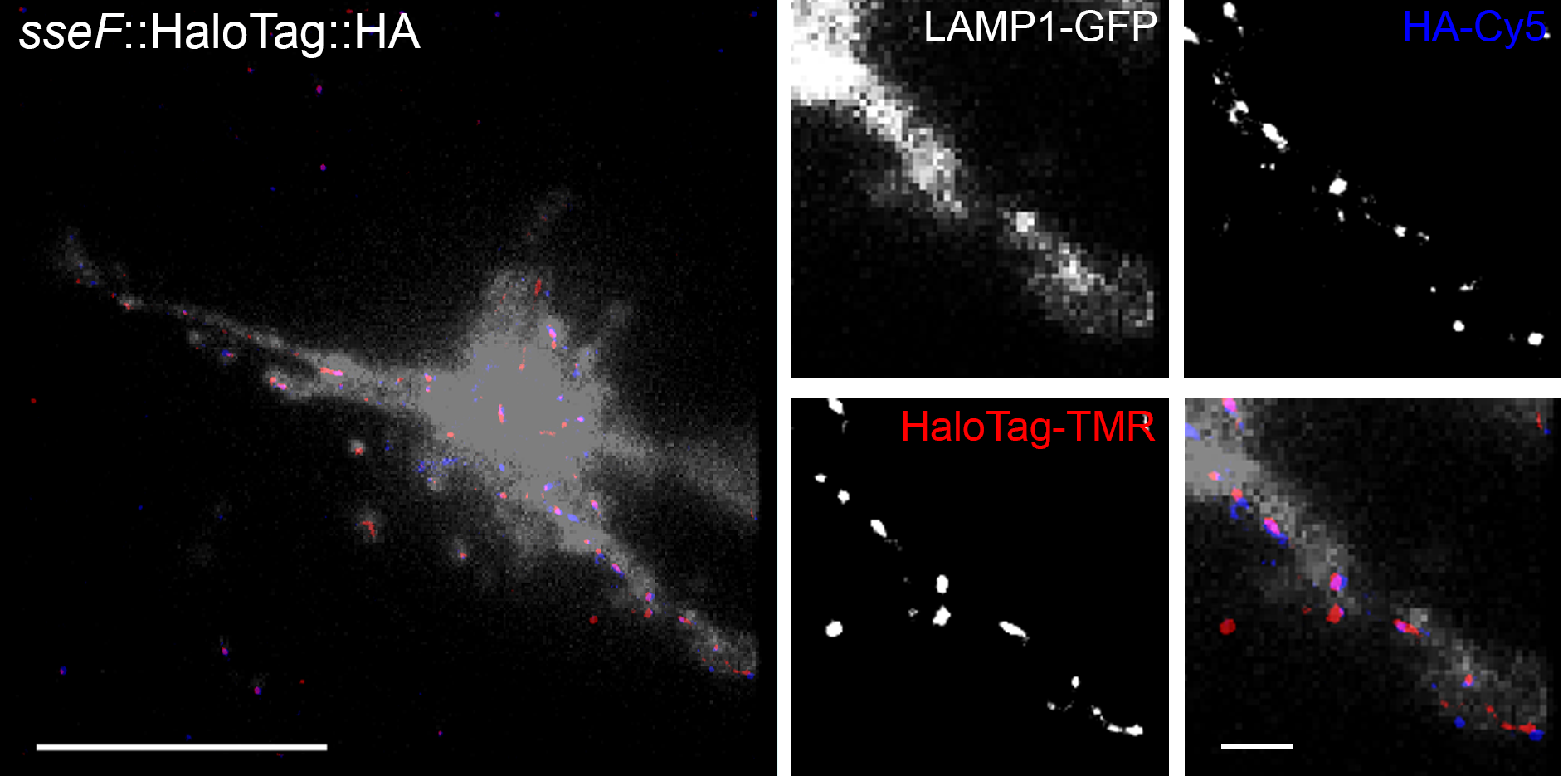

Supplement: FIG S7 [file mBio.00769-19-sf007.tif]
